# Correlation of the Rates of Solvolysis of Two Arenesulfonyl Chlorides and of *trans*-β-Styrenesulfonyl Chloride – Precursors in the Development of New Pharmaceuticals

**DOI:** 10.3390/ijms9122639

**Published:** 2008-12-17

**Authors:** Zoon Ha Ryu, Sang Wok Lee, Malcolm J. D’Souza, Lamia Yaakoubd, Samantha E. Feld, Dennis N. Kevill

**Affiliations:** 1 Department of Chemistry, Dong-Eui University, 24 Kaya-dong, Busan-Jin-Gu, Busan 614-714, Korea; 2 Department of Chemistry, Wesley College, 120 N. State Street, Dover, Delaware 19901-3875, USA; 3 Department of Chemistry and Biochemistry, Northern Illinois University, DeKalb, Illinois 60115-2862, USA

**Keywords:** Solvolysis, sulfonyl transfer, benzenesulfonyl chloride, *para*-nitrobenzenesulfonyl chloride, *trans*-β-styrenesulfonyl chloride, Grunwald-Winstein equation, Linear Free Energy Relationships, kinetic solvent isotope effect, general base catalysis

## Abstract

Additional specific rates of solvolysis have been determined, mainly in fluoroalcohol containing solvents, for benzenesulfonyl chloride (**1**) and *p*-nitrobenzene-sulfonyl chloride (**2**). For *trans*-β-styrenesulfonyl chloride (**3**), a study has been carried out in 43 pure and binary solvents, covering a wide range of hyroxylic solvent systems. For the specific rates of solvolyses of each of the three substrates, a good correlation was obtained over the full range of solvents when the extended Grunwald-Winstein equation was applied. The sensitivities to changes in solvent nucleophilicity and solvent ionizing power are similar to values determined earlier and an S_N_2 process is proposed. For **3**, kinetic solvent isotope effects of 1.46 for *k*_H_2_O_/*k*_D_2_O_ and 1.76 for *k*_MeOH_/*k*_MeOD_ were determined. These are also compared to literature values for other sulfonyl chlorides.

## 1. Introduction

The syntheses of biologically active sulfonamides usually involve nucleophilic attack of an amine on a sulfonyl chloride, such as benzenesulfonyl chloride (**1**) or *p*-nitrobenzenesulfonyl chloride (**2**). Several thousand sulfonamides have been evaluated in terms of biological activity and this synthesis area remains a very active one. For example, within the last two years (2007-2008) over fifty patent applications have been filed relating to the utility of sulfonamides synthesized from *trans*-β-styrene-sulfonyl chloride [(*E*)-2-phenylethenesulfonyl chloride, **3**].

Despite the widespread application of nucleophilic substitution reactions of sulfonyl chlorides, there has been considerable uncertainty regarding the detailed mechanism of the process [1]. Such knowledge would be useful in the selection of optimum reaction conditions. Solvolytic reactions afford a good prototype for these substitutions [2].

The original Grunwald-Winstein equation was developed in 1948 [3] so as to give a linear free energy relationship treatment of the specific rates (first-order rate coefficients) for solvolysis of initially neutral substrates reacting by an ionization (S_N_1 + E1) mechanism. The equation can be

(1) $$\text{log}\;(k/k_{o})\;=\;mY\;+\;c\;(1)$$

expressed as in equation 1 where *k* and *k_o_* are the specific rates of solvolysis in a given solvent and in the standard solvent (arbitrary chosen as 80% ethanol), respectively, *m* represents the sensitivity to changes in the solvent ionizing power *Y* (arbitrary set at unity for *tert*-butyl chloride solvolyses in the early studies), and *c* is a constant (residual) term. It has been demonstrated [3] that different *Y* scales are needed for different leaving groups and that the use of adamantyl derivatives minimizes nucleophilic solvation effects at the α-carbon, leading to a purer ionization process than for *t*-butyl chloride.

For bimolecular (S_N_2 and/or E2) reactions, in which the solvent also acts as a nucleophile or base, the correlation can be extended [4] to include a term governed by the sensitivity (*l*) to changes in solvent nucleophilicity (*N*), as shown in equation 2.

(2) $$\text{log}\;(k/k_{o})\;=\;lN\;+\;mY\;+\;c\;(2)$$

Initially, methyl *p*-toluenesulfonate was used as the standard substrate to arrive at a solvent nucleophilicity scale termed *N*_OTs_ [5]. This required making an estimate of the sensitivity of the solvolysis of methyl *p*-toluenesulfonate towards changes in solvent ionizing power (the *m* value of equation 2), which cannot be obtained directly. More recently, to minimize leaving group effects upon specific rate variation as the solvent is varied, a scale, labeled *N*_T_, has been developed [6] based upon the solvolyses of the *S*-methyldibenzothiophenium ion. In these solvolyses, the leaving group is a neutral molecule and the appreciable solvent-leaving group interaction, when the leaving group is anionic, is avoided. The development of solvent nucleophilicity scales has been reviewed [2, 7].

It is generally accepted that, for most sulfonyl chlorides, the mechanism of solvolysis is bimolecular in character [1, 2, 8–22]. There have, however, been claims of S_N_1 (ionization) reactions for a few substrates. The solvolyses of *N*,*N*-dimethylsulfamoyl chloride were considered by Hall [8] to be unimolecular, and this view was supported by Robertson [23].

Convincing evidence has been presented that the solvolysis are S_N_2 in character [21, 24, 25]. Similarily, a claim that reactions of alkanesulfonyl chlorides solvolyze, at least in part, by S_N_1 reactions in polar solvents [26] is no longer tenable [1, 2]. Also, a claim that 2,4,6-trimethylbenzene-sulfonyl chloride solvolyzes by the S_N_1 mechanism [16, 27, 28] has been shown to be incorrect [1, 2, 17, 18]. At the present time, there does not seem to be any convincing evidence for S_N_1 reaction in the solvolysis of a sulfonyl chloride.

Although the solvolyses are established as bimolecular, other more detailed aspects remain to be considered. There has been considerable discussion as to whether the mechanism involves a concerted S_N_2 pathway or an addition-elimination (S_A_N) pathway and, irrespective of which of these pathways operates, whether attack by one solvent molecule is assisted by a general base catalysis from the involvement of a second solvent molecule. The S_A_N mechanism has largely been proposed for reactions with added nucleophiles under what otherwise would be solvolytic conditions [1] and the mechanism has only occasionally been suggested for solvolytic reactions. In proposing a duality of reaction channels for solvolyses of electron-rich benzenesulfonyl chlorides, it was suggested that one channel involves S_N_2 and the other S_A_N or a base catalyzed S_N_2 reaction [18]. Subsequent publication by the authors, favor the base catalyzed S_N_2 reaction as the second channel [29, 30], without totally ruling out the possibility of the addition-elimination pathway [31].

**Scheme 1:**
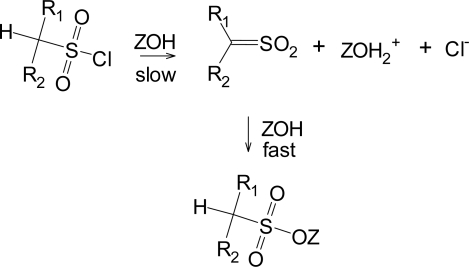
Formation of a sulfene intermediate from substrates having a hydrogen plus electron-withdrawing groups on the α-carbon.

Although the addition-elimination pathway appears to be disfavored for sulfonyl chlorides, the elimination-addition reaction (Scheme 1) is well established for suitable substrates, primarily those which have a hydrogen plus electron-withdrawing groups on the α-carbon [11, 32]. The sulfene intermediate formed in the slow step then readily adds solvent (ZOH) to give (in the absence of isotopic labeling) a product identical to the direct substitution product.

**Scheme 2:**
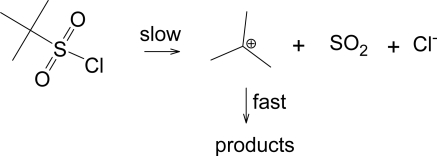
Solvolysis-decomposition pathway for *tert*-butylsulfonyl chloride.

Another pathway has been observed when the R group of RSO_2_Cl forms a relatively stable carbocation, such as a *tertiary*-butyl group [33]. This pathway shown in Scheme 2, leads to the products typically formed from a tertiary carbocation, including *tert*-butyl chloride. This solvolysis-decomposition reaction is closely related to that observed [34] with similarily constituted tert-alkyl chloroformates (ROCOCl), but with loss of SO_2_ rather than CO_2_.

The three substrates of the present study would not be expected to proceed by the ionization, elimination-addition, or solvolysis-decomposition routes and one can concentrate on whether the rate-determining nucleophilic attack by the solvent involves a concerted or stepwise substitution pathway, and on whether general base catalysis is assisting the process.

## 2. Results and Discussion

**Scheme 3:**
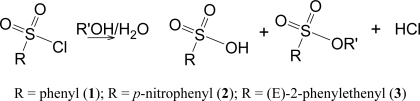
Solvolysis for benzenesulfonyl chloride (**1**), *p*-nitrobenzenesulfonyl chloride (**2**), and *trans*-β-styrenesulfonyl chloride [(E)-2-phenylethenesulfonyl chloride] (**3**).

The solvolyses lead to the shown products (Scheme 3) at rates which are convenient for measurement by the titration of the developed acid. Both the sulfonic acid and the hydrochloric acid titrate as strong acids.

The specific rates already available for **1** and **2** in the literature were complemented by the determination of additional values. For **1** values were already available in aqueous acetone [35], aqueous dioxane [28], water [28, 36], and 97% 2,2,2-trifluoroethanol (TFE) [22]. Values were also available for ethanol and mixtures of ethanol with water or TFE [37] but a repeat of these determinations led to specific rates some 20 - 35% lower; the lower values were used in the correlations. Additional values were obtained in aqueous methanol, 80% acetone, 80 – 50% TFE, TFE-ethanol, and aqueous 1,1,1,3,3,3-hexafluoro-2-propanol (HFIP). All of the specific rate values at 35.0 °C for solvolyses of **1**, as used in the correlation, are shown in Table 1.

**Figure 2.**
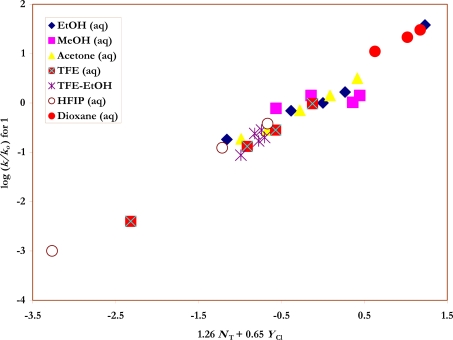
The plot of log (*k/k_o_*) vs. (1.26 *N_T_* + 0.65 *Y_Cl_*) for the solvolyses of benzene-sulfonyl chloride (**1)** in pure and binary solvents at 35.0 ºC.

**Figure 3.**
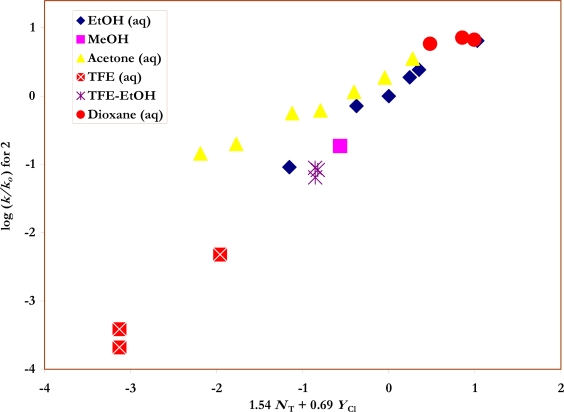
The plot of log (*k/k_o_*) vs. (1.54 *N_T_* + 0.69 *Y_Cl_*) for the solvolyses of *p*-nitro-benzenesulfonyl chloride (**2**) in pure and binary solvents at 35.0 ºC. Four acetone-water points (90% - 75%) were not included in the correlation but are added to show the extent of their deviations from the plot.

Also shown in Table 1 are the specific rates of solvolysis for **2** used in the correlations. Values were available for ethanol [37], water [28] and their mixtures [37], for several aqueous acetone [35] and aqueous dioxane [28] compositions, and for TFE-ethanol mixtures [37]. Additional determinations were made in methanol, 80% ethanol, 90% and 80% acetone, and in 100%, 97% and 90% TFE. The *N*_T_ [6, 7] and *Y*_Cl_ [3, 38, 39] values needed for the application of equation 2 to the specific rate values for **1** and **2** (Figures 2 and 3) are also listed in the Table.

**Figure 4.**
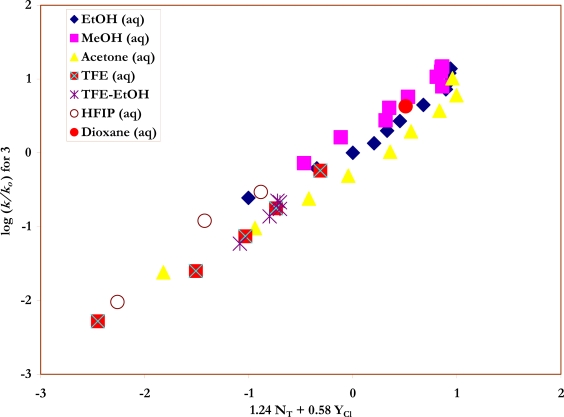
The plot of log (*k/k_o_*) vs. (1.24 *N_T_* + 0.58 *Y_Cl_*) for the solvolyses of *trans*-β-styrenesulfonyl chloride (**3**) in pure and binary solvents at 45.0 ºC.

Specific rates of solvolysis of *trans*-β-styrenesulfonyl chloride (**3**) have not been previously determined and a study was carried out, at 45.0 °C, in terms of increases in conductivity observed as the strong acids are produced (Scheme 3) in ethanol, methanol, and water, and the full range of aqueous ethanol and methanol compositions. These values are reported in Table 2. A value obtained in deuterium oxide could be combined with the water value to give a kinetic solvent isotope effect (*k*_H_2_O_/*k*_D_2_O_) of 1.46 ± 0.02. Similarly the values in methanol and methanol-*d* give a KSIE value (*k*_MeOH_/*k*_MeOD_) of 1.76 ± 0.02. In Table 3, two additional *N*_T_ values and one additional *Y*_Cl_ value for dioxane-water mixtures are abstracted from the literature [18, 40, 41] and specific rates are tabulated for a series of aqueous acetone and aqueous dioxane compositions. Table 4 contains the specific rates obtained in TFE-water, HFIP-water, and TFE-ethanol. The ratio of the specific rates in 40% ethanol (Table 2) and 97% TFE (Table 4), two solvents of similar ionizing power but very different solvent nucleophilicities, (*k*_40EtOH_/*k*_97TFE_) is 846, indicating that solvent nucleophilicity is an important rate-controlling factor [3, 22, 42]. The correlation of the specific rates of solvolysis of **3**, in all 43 solvents and using the extended Grunwald-Winstein equation, is presented in Figure 4.

For nine representative solvents, specific rates were also determined at 25.0, 35.0, and 55.0 °C (Table 5) and the determinations at the four temperatures (45.0 °C values from Tables 2–4) were analyzed using the Eyring equation, so as to obtain enthalpies and entropies of activation. The entropies of activation were all appreciably negative (-13 to -34 k cal mol^-1^ K^-1^), consistent with a bimolecular (or higher molecularity) solvolytic process.

The new values for the specifc rates of solvolysis of **1** and **2**, in conjunction with earlier values, allow for correlations using equation 2 for a good selection of solvent types, including the important fluoroalcohol solvents. Similarly, within this study, the specific rates of solvolysis of **3** have been determined in 43 well chosen solvents. The sensitivity values, the residual (intercept) value, and the associated standard errors are reported within Table 6, together with the multiple correlation coefficient and the *F*-test values. With **1** and **3**, all of the available values are included in the correlation. For **2**, (Figure 3) it is found that two data points, for 90% and 87% acetone, lie appreciably off the correlation line and two data points (80% and 75% acetone) lie somewhat off the plot. In the table, correlation values are given for all solvents and also with the two and four data points obtained in the acetone-rich solvents omitted. The goodness-of-fit parameters show a progressive improvement for the limited analyses. Since the specific rate in 87% acetone is from an independent source [35] and the values for 90% and 87% are consistent with each other, the deviations appear to be real and not due to experimental error. The observation that these deviations appear only when the *p*-nitro group is present suggests a specific interaction between the group and the acetone-rich solvents. It has previously been observed that the Hammett σ values for a *p*-nitro group are somewhat dependent on the solvent [43, 44]. The benefits of a good mix of solvent type are well illustrated by the previous approximate *l* and *m* values [20] for 16 solvents for the solvolyses of **2** of 1.39±0.50 for *l*, and 0.65±0.13 for *m* improving to values of 1.44±0.11 and 0.57±0.06 for the full 23 solvents now available (Table 6).

Also included in Table 6 are the corresponding sensitivity and goodness-of-fit parameters from previous correlations of sulfonyl chlorides [45] using the extended Grunwald-Winstein equation. With the exception of low values for both *l* and *m* for the solvolyses of *α*-toluenesulfonyl chloride, the *l* values vary from a low of 1.07±0.08 for *p*-methoxybenzenesulfonyl chloride to a value of 1.54±0.07 for **2**, with the values for **1** (1.26±0.05) and **3** (1.24±0.04) being almost identical and towards the middle of the range. The *l*/*m* ratios are very close in value for all of the entries in the table, varying from a low of 1.67 for *N*,*N*-dimethylsulfamoyl chloride to 2.39 to methanesulfonyl chloride. Restricting to arenesulfonyl chlorides, the lowest value is 1.78 for *p*-MeOC_6_H_4_SO_2_Cl and the highest is 2.23 for *p*-NO_2_C_6_H_4_SO_2_Cl. The *l* value and the *l*/*m* value variations for the arenesulfonyl chlorides are consistent with a situation in which all members of the family solvolyze by an S_N_2 mechanism but with the detailed pathways involving a looser transition state in the presence of the electron supplying *p*-MeO group, relative to **1**, and varying to a tighter transition state with the electron-withdrawing *p*-NO_2_ group as the substituent [37, 46].

A major consideration over the past two decades involves the extent to which the bimolecular nucleophilic substitution reactions of sulfonyl chlorides may be proceeding with general base catalysis. Evidence comes mainly from two sources: the successful analysis in binary water-alcohol solvents in terms of the four possible specific rates expected to contribute if the pathway is termolecular (first-order in substrate and second-order in “solvent” (alcohol and/or water), as was observed for the solvolyses of **2** [30], and consideration of the magnitude of KSIEs, using the rationale that higher values would be expected with the involvement of two solvent molecules (as general base catalysis and as nucleophile) compared to the involvement of only one as nucleophile [18, 19, 29, 31, 47–49]. Initially, water and deuterium oxide were used as the solvents [47] but solubility problems can arise with organic substrates and methanol and methanol-*d* (MeOD) have been found to afford a useful alternative [19]. The KSIE values are usually slightly higher in value in the methanol solvents [19, 31, 49] but the solvolyses of methanesulfonyl chloride provide an exception to this generalization [48].

**Scheme 4:**
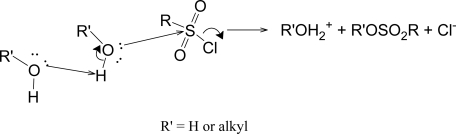
Substitution being assisted by general base catalysis.

The KSIE values (*k*_MeOH_/*k*_MeOD_) for **1**-**3**, are tabulated in Table 7, where they are compared with values for other sulfonyl chlorides. The values are all appreciably above unity (1.45 to 2.54), consistent with the nucleophilic attack by solvent indicated by the treatment using equation 2. The monosubstituted arenesulfonyl chlorides have KSIEs which vary from 1.58 for the *p*-MeO-derivative to 1.72 for the *p*-Me-derivative to 1.79 for the parent benzenesulfonyl chloride (**1**) to 2.31 for the *p*-NO_2_-derivative (**2**) [19]. These increases parallel increases in the *k*_40EtOH_/*k*_97TFE_ ratio (a simplified treatment for determining the sensitivity to solvent nucleophilicity, which works well provided there is no intervening change in mechanism), with values of 300, 450, 2900, and 15000, respectively. That care must be taken in interrelating the two types of entry in Table 7 is indicated, however, by very similar (*k*_40EtOH_/*k*_97TFE_) ratios (2010 and 2790) for MeSO_2_Cl [49,50] and *i*-PrSO_2_Cl [21], but very different (*k*_MeOH_/*k*_MeOD_) ratios of 1.62 and 2.54 at 25 oC and 1.51 and 2.41 at 35 oC, respectively. It has been proposed that the KSIE values in Table 7 are sufficiently large to justify the proposal of the substitution reactions being assisted by general base catalysis (Scheme 4) [48, 49]. For a series of *para*-substituted benzenesulfonyl chlorides, it has been suggested that both assisted and unassisted S_N_2 pathways operate, with the presence of an electron-withdrawing substituent strongly favoring the pathway assisted by general-base catalysis [19]. At the other extreme, it has been proposed that, in TFE [17] or in highly ionizing aqueous acetic acid [51], the solvolyses of *p*-(dimethylamino)benzenesulfonyl chloride occur through a concerted bimolecular mechanism with bond breaking running ahead of bond formation in the transition state. A problem with the evaluation of the relevance of general-base catalysis effects in the solvolyses of sulfonyl chlorides is that, although the possibility of analyzing in terms of third-order rate coefficients in the solvolyses of *para*-nitrobenzenesulfonyl chloride (**2**) is suggestive [30], there are no substrates for which one can say with a high degree of confidence that the bimolecular solvolyses are either assisted or unassisted by general base catalysis. This leads to a situation in which there are no firmly established reference (standard) KSIE values for solvolyses involving nucleophilic attack at the sulfur atom of a sulfonyl chloride.

## 3. Conclusions

Additional specific rates of solvolysis, mainly in solvents rich in fluoroalcohol, have been determined at 35.0 °C for benzenesulfonyl chloride (**1**) and for *p*-nitrobenzenesulfonyl chloride (**2**). Correlations, using equation 2, previously carried out with 15 or 16 solvents [20] and handicapped by a shortage of solvents rich in fluoroalcohol, are considerably improved in the present study, with 29 and 23 solvents, respectively, now available. For **2**, there is noticeable deviation from the correlation line for solvents rich in acetone and it is suggested that this could be a consequence of specific interactions between the nitro-group of **2** and the solvent.

A comprehensive study of the specific rates of solvolysis of *trans*-β-styrenesulfonyl chloride (**3**) in 43 solvents showed that a very good extended Grunwald-Winstein equation treatment of the data (equation 2) can be carried out with all solvents included. Values typical for solvolyses of sulfonyl chlorides (*l* = 1.24; *m* = 0.58) were obtained. The KSIEs (at 45.0 °C) of 1.76 in methanol and methanol-*d* and of 1.46 in H_2_O and D_2_O are also typical values for the solvolyses of sulfonyl chlorides.

Indeed, a comparison (Table 6) of the results from the correlations using equation 2, for all sulfonyl chlorides studied in this way leads, with one exception, to *l* values in the range 1.07 – 1.54 and *m* values in the range of 0.49 – 0.72. The one exception, *α*-toluenesulfonyl chloride, has low values for both *l* and *m*, which lead to a typical *l*/*m* ratio of 2.05 (range of value 1.67 – 2.53).

It appears that all of the sulfonyl chlorides so far investigated in terms of equation 2 are solvolyzing by a common mechanism, generally believed to be S_N_2. In support of a bimolecular pathway, very recent calculations [52] indicate that most sulfonyl chlorides solvolyze with relatively little development of cationic character. The trends of values seen for *l*, *m*, and the *l*/*m* ratio (Table 6) plus the trends in the *k*_40EtOH_/*k*_97TFE_ ratio (a simplified measure of the sensitivity towards changes in solvent nucleophilicity) of Table 7 and, to some extent, the trends in KSIE values for solvolyses in methanol and methanol-*d* support a mechanism with variations in detailed transition state structure within a conventional S_N_2 process and/or variations in the extent of general base catalysis (Scheme 4) towards a fundamentally S_N_2 process.

## 4. Experimental Section

The benzenesulfonyl chloride (**1**, Sigma-Aldrich, 99%), *p*-nitrobenzenesulfonyl chloride (2, Sigma-Aldrich, 97%), and *trans*-β-styrenesulfonyl chloride (3, Sigma-Aldrich, 97%) were used as received. Solvents were purified as described previously [6]. The specific rates of solvolysis of **1** and **2** were determined by titration of the acid developed [6]. The calculation of the specific rates of solvolysis (first-order rate coefficients) were obtained when the conventional Guggenheim treatment [53] was modified [54] so as to give the infinity titer, which was then used to calculate for each run a series of integrated rate coefficients. For faster reactions, experimental values for the infinity titer could be obtained and these were in good agreement with the estimated values. The specific rates and associated standard deviations, as presented in Table 1, are obtained by averaging all of the values from, at least, duplicate runs. For the solvolysis of **3**, the specific rates were determined using an apparatus that allows a rapid response to changes in conductivity [55]. The details of the apparatus have been reported previously [56]. Typical, the determinations followed the injection of 4 μL of a 0.5 to 3.0% (w/w) solution of the substrate in dry acetonitrile into 2 mL of the appropriate solvent. Specific rates, with associated standard deviations, were determined [53] from a computer analysis (Guggenheim Method) of the plots of conductivity against time. The multiple regression analyses were carried out using commercially available statistical packages.

## Figures and Tables

**Figure 1.**
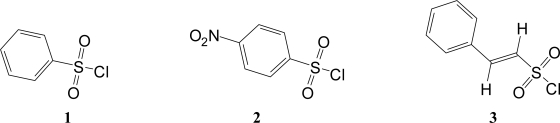
Molecular structures of benzenesulfonyl chloride (**1**), *p-*nitrobenzenesulfonyl chloride (**2**), and *trans*-β-styrenesulfonyl chloride (**3**).

**Table 1.** Specific rates of solvolysis (*k*) of benzenesulfonyl chloride (**1**) and *p*–nitrobenzenesulfonyl chloride (**2**) in pure and binary solvents at 35.0 ºC and the solvent nucleophilicity (*N*_T_) and solvent ionizing power (*Y*_Cl_) values for the solvents.

Solvent (%)*^a^*	1; 10^5^*k*(s^−1^)*^b^*	2; 10^5^*k*(s^−1^)*^b^*	*N*_T_*^c^*	*Y*_Cl_*^d^*
100% EtOH	3.60±0.34*^e^*	(10.7)*^f^*	0.37	−2.52
90% EtOH	13.6±0.6*^e^*	(83.9)*^f^*	0.16	−0.94
80% EtOH	19.6±1.1*^e^*	117±5*^e^*	0.00	0.00
70% EtOH	32.4±1.2*^e^*	(221)*^f^*	−0.20	0.78
60% EtOH		(285)*^f^*	−0.39	1.38
100% H_2_O	(751)*^g^* (756)*^h,^^i^*	(759)*^h^*	−1.38	4.57
100% MeOH	15.1±0.8	21.8±1.4	0.17	−1.17
90% MeOH	27.9±1.4		−0.01	−0.18
80% MeOH	20.1±0.8		−0.06	0.67
70% MeOH	27.6±0.9		−0.40	1.46
90% Acetone		16.9±0.7	−0.35	−2.39
87% Acetone		(23.3)*^j^*	−0.34	−1.81
80% Acetone	3.64±0.20	65.7±2.9	−0.37	−0.83
75% Acetone	(5.57)*^j^*	(72.1)*^j^*	−0.39*^k^*	−0.28 *^k^*
63% Acetone	(14.0)*^j^*	(135)*^j^*	−0.54*^k^*	0.62*^k^*
52% Acetone	(27.9)*^j^*	(219)*^j^*	−0.68*^k^*	1.45*^k^*
42% Acetone	(62.5)*^j^*	(414)*^j^*	−0.81*^k^*	2.21*^k^*
34% Dioxane	(216)*^h^*	(686)*^h^*	−0.92*^k^*	2.75*^k^*
18% Dioxane	(419)*^h^*	(844)*^h^*	−1.13*^k^*	3.76*^k^*
10% Dioxane	(592)*^h^*	(786)*^h^*	−1.23*^k^*	4.23*^k^*
100% TFE		0.0247±0.0011	−3.93	2.81
97% TFE (w/w)	(0.0774)*^l^*	0.0450±0.0018*^m^*	−3.30	2.83
90% TFE (w/w)		0.562±0.021	−2.55	2.85
80% TFE (w/w)	2.57±0.09		−2.19	2.90
70% TFE (w/w)	5.48±0.22		−1.98	2.96
50% TFE (w/w)	19.0±0.5		−1.73	3.16
80T-20E	1.70±0.05		−1.76	1.89
60T-40E	3.33±0.08		−0.94	0.63
50T-50E	3.88±0.18		−0.64	0.16
40T-60E	5.48±0.21	(7.61)*^f^*	−0.34	−0.48
30T-70E		(9.84)*^f^*	−0.11*^k^*	−0.95*^k^*
20T-80E	4.73±0.10*^e^*	(10.5)*^f^*	0.08	−1.42
97% HFIP(w/w)	0.0196±0.0011		−5.26	5.17
70% HFIP(w/w)	2.40±0.06		−2.94	3.83
50% HFIP(w/w)	7.52±0.22		−2.49	3.80

a On volume-volume basis at 25.0 ºC, except when indicated as weight-weight (w/w) basis.

b With associated standard deviations; values in parentheses are obtained (directly or by an Arrhenius treatment of specific rates) from the literature, as indicated.

c From ref. [7].

d From refs. [3, 38, 39].

e These values are 20-35% lower than values reported in ref. [37].

f From ref. [37].

g From Arrhenius treatment of values in ref. [36].

h Calculated using the activation parameters reported in ref. [28].

i A value of 825 is calculated from the rate data of ref. [10].

j These values are calculated from specific rates reported in ref. [35].

k Obtained by interpolation.

l Estimated from values in ref. [22].

m Arrhenius equation treatment of specific rates in ref. [22] leads to a value of 0.0411.

**Table 2.** Specific rates of solvolysis (*k*) of *trans*-β-styrenesulfonyl chloride (**3**)*^a^* in pure ethanol, methanol, and water, and in alcohol-water binary mixtures at 45.0 ºC.

Solvent*^b^*	*k*(s^−1^)	n*^c^*	*N*_T_*^d^*	*Y*_Cl_*^d^*
100 EtOH	(2.18±0.03)×10^−4^	3	0.37	−2.52
90 EtOH	(5.40±0.01)×10^−4^	1	0.16	−0.94
80 EtOH	(8.83±0.02)×10^−4^	1	0.00	0.00
70 EtOH	(1.20±0.01)×10^−3^	1	−0.20	0.78
60 EtOH	(1.76±0.01)×10^−3^	1	−0.38	1.38
50 EtOH	(2.40±0.01)×10^−3^	1	−0.58	2.02
40 EtOH	(3.95±0.02)×10^−3^	1	−0.74	2.75
30 EtOH	(6.35±0.01)×10^−3^*^e^*	1	−0.93	3.53
20 EtOH	(8.23±0.03)×10^−3^*^e^*	1	−1.16	4.09
10 EtOH	(1.07±0.07)×10^−2^*^f^*	4	−1.31	4.40
H_2_O	(1.21±0.05)×10^−2^*^f^*	6	−1.38	4.57
D_2_O*g*	(8.28±0.20)×10^−3^*^f^*	5		
100 MeOH	(6.33±0.08)×10^−4^	5	0.17	−1.17
100 MeOD*^h^*	(3.59±0.08)×10^−4^	4		
90 MeOH	(1.44±0.01)×10^−3^	1	−0.01	−0.18
80 MeOH	(2.43±0.01)×10^−3^	3	−0.06	0.67
70 MeOH	(3.61±0.01)×10^−3^	1	−0.40	1.46
60 MeOH	(5.12±0.01)×10^−3^	1	−0.54	2.07
50 MeOH	(7.04±0.01)×10^−3^	1	−0.57	2.70
40 MeOH	(9.43±0.01)×10^−3^	3	−0.87	3.25
30 MeOH	(1.10±0.01)×10^−2^*^e^*	1	−1.06	3.73
20 MeOH	(1.29±0.01)×10^−2^*^f^*	1	−1.23	4.10
10 MeOH	(1.31±0.01)×10^−2^*^f^*	1	−1.36	4.39

a Determined conductimetrically and typically injected 4 μL of 3% (w/w) substrate in dry acetonitrile into the kinetic apparatus containing 2 mL of solvent (concentration of *ca*. 2.4 × 10^−4^ mol dm^−3^); errors accompanying the specific rates are standard deviations.

b Percentage by volume at 25.0 ºC of organic component (v/v%).

c Number of runs.

d From listings in ref.[7] for *N*_T_ and in refs. [3, 38, 39] for *Y*_Cl_.

e Injected 4 μL of 1.0% (w/w) substrate in dry acetonitrile (concentration of *ca*. 8 × 10^−5^ mol dm^−3^).

f Injected 4 μL of 0.5% (w/w) substrate in dry acetonitrile (concentration of *ca*. 4 × 10^−5^ mol dm^−3)^.

g Kinetic Solvent Isotope Effect (KSIE), *k*_H_2_O_/*k*_D_2_O_ = 1.46 (±0.02) at 45.0 ºC (average from five paired values).

h KSIE, *k*_MeOH_/*k*_MeOD_ = 1.76 (±0.02) at 45.0 ºC (average from four paired values).

**Table 3.** Specific rates of solvolysis (*k*) of *trans*-β-styrenesulfonyl chloride (**3**)*^a^* in aqueous acetone and aqueous dioxane at 45.0 ºC.

Solvent*^b^*	*k*(s^−1^)	n*^c^*	*N*_T_*^d^*	*Y*_Cl_*^d^*
90 Acetone	(2.12±0.01)×10^−5^	1	−0.35	−2.39
80 Acetone	(8.53±0.01)×10^−5^	1	−0.37	−0.83
70 Acetone	(2.10±0.01)×10^−4^	1	−0.42	0.17
60 Acetone	(4.33±0.05)×10^−4^	1	−0.48	0.95
50 Acetone	(9.14±0.01)×10^−4^	1	−0.52	1.73
40 Acetone	(1.74±0.02)×10^−3^	1	−0.70	2.46
30 Acetone	(3.29±0.01)×10^−3^*^e^*	1	−0.83	3.21
20 Acetone	(5.36±0.01)×10^−3^*^e^*	1	−0.96	3.77
10 Acetone	(9.01±0.06)×10^−3^*^f^*	3	1.23	4.28
80 Dioxane	(7.58±0.01)×10^−5^	1	−0.46	
50 Dioxane	(1.13±0.01)×10^−3^	1	−0.66*^g^*	
30 Dioxane	(3.75±0.04)×10^−3^*^e^*	1	−0.98*^h^*	2.97*^i^*

a-f See footnotes to Table 2.

g From ref. [40].

h From ref. [41].

i From ref. [18].

**Table 4.** Specific rates of solvolysis (*k*) of *trans*-β-styrenesulfonyl chloride (**3**)*^a^* in binary mixtures of water with 2, 2, 2-trifluoroethanol (TFE) and 1, 1, 1, 3, 3, 3-hexafluoro-2-propanol (HFIP) and in TFE-ethanol (T-E) mixtures at 45.0 ºC.

Solvent*^b^*	*k*(s^−1^)	n*^c^*	*N*_T_*^d^*	*Y*_Cl_*^d^*
97 TFE*^e^*	(4.67±0.01)×10^−6^	1	−3.30	2.83
90 TFE	(2.22±0.01)×10^−5^	1	−2.55	2.85
80 TFE	(6.53±0.01)×10^−5^	1	−2.19	2.90
70 TFE	(1.57±0.01)×10^−4^	1	−1.98	2.96
50 TFE	(5.12±0.07)×10^−4^	2	−1.73	3.16
90 HFIP	(8.33±0.02)×10^−6^	1	−3.84	4.31*^f^*
70 HFIP	(1.06±0.01)×10^−4^	1	−2.94	3.83*^f^*
50 HFIP	(2.61±0.01)×10^−4^	1	−2.49	3.80
80T-20E*^g^*	(5.18±0.02)×10^−5^	1	−1.76	1.89
60T-40E*^g^*	(1.21±0.01)×10^−4^	1	−0.94	0.63
50T-50E*^g^*	(1.53±0.01)×10^−4^	2	−0.64	0.16
40T-60E*^g^*	(1.89±0.01)×10^−4^	1	−0.34	−0.48
20T-80E*^g^*	(1.97±0.02)×10^−4^	2	0.08	−1.42

a See footnote a in Table 2.

b Unless otherwise stated, percentage by weight of organic component (w/w%).

c Number of runs.

d See footnote d in Table 2.

e Ratio specific rates, (k_40EtOH_/k_97TFE_), in 40 EtOH and 97TFE = 846.

f From ref. [38].

g Percentages by volume at 25.0 °C.

**Table 5.** Specific rates (*k/s*^−1^) of solvolysis for *trans*-β-Styrenesulfonyl Chloride at temperature other than 45.0 ºC and activation parameters.

Solvent	T, ºC	*k*/s^−1^	ΔH^≠^_298_/(kcal/mol)*a*	ΔS^≠^_298_/(cal/mol K)*a*
100 EtOH	25	(2.72±0.02)×10^−5^	19.0±0.2	−15.9±0.8
	35	(8.32±0.01)×10^−5^		
	55	(5.64±0.01)×10^−4^		
80 EtOH	25	(1.47±0.03)×10^−4^	16.0±0.3	−22.3±1.0
	35	(3.51±0.01)×10^−4^		
	55	(1.88±0.03)×10^−3^		
30 EtOH	25	(1.25±0.04)×10^−3^	14.3±0.2	−23.8±0.9
	35	(2.94±0.01)×10^−3^		
	55	(1.25±0.04)×10^−2^		
100 MeOH	25	(1.14±0.01)×10^−4^	14.9±0.5	−26.6±1.6
	35	(2.67±0.01)×10^−4^		
	55	(1.22±0.01)×10^−3^		
70 MeOH	25	(6.84±0.01)×10^−4^	15.0±0.1	−22.7±0.4
	35	(1.65±0.04)×10^−3^		
	55	(7.66±0.04)×10^−3^		
50 HFIP	25	(3.65±0.02)×10^−5^	17.9±0.2	−18.8±0.8
	35	(9.61±0.01)×10^−5^		
	55	(6.27±0.01)×10^−4^		
80 Acetone	25	(1.77±0.01)×10^−5^	14.0±0.4	−33.4±1.4
	35	(4.30±0.02)×10^−5^		
	55	(1.71±0.01)×10^−4^		
80 Dioxane	25	(1.52±0.01)×10^−4^	14.8±0.3	−31.0±0.9
	35	(3.35±0.03)×10^−5^		
	55	(1.63±0.01)×10^−4^		
30 Dioxane	25	(4.98±0.01)×10^−4^	17.9±0.3	−13.4±0.9
	35	(1.41±0.01)×10^−3^		
	55	(8.66±0.01)×10^−3^		

a Obtained from an Eyring plot and using also the specific rate at 45.0 ºC from Table 2, 3, or 4; with associated standard errors.

**Table 6.** Correlation of the specific rates of solvolytic nucleophilic displacement at the sulfur of sulfonyl chlorides using the extended Grunwald-Winstein equation (equation 2)

Substrate	T ºC	n*^a^*	*l^b^*	*m^b^*	*c*	*R^c^*	*F^d^*	*l/m*
**1**	35.0	29	1.26±0.05	0.65±0.03	0.13±0.05	0.979	304	1.94
**2**	35.0	23	1.44±0.11	0.57±0.06	0.20±0.11	0.945	83	2.53
		21*^e^*	1.52±0.09	0.66±0.05	0.10±0.09	0.968	134	2.30
		19*^f^*	1.54±0.08	0.69±0.04	0.01±0.08	0.981	209	2.23
**3**	45.0	43	1.24±0.04	0.58±0.02	0.07±0.04	0.982	542	2.14
*p*-MeOC_6_H_4_SO_2_Cl	25.0	38*^g^*	1.07±0.08	0.60±0.03	0.22±0.06	0.967	254	1.78
*p*-MeC_6_H_4_SO_2_Cl	25.0	34*^g^*	1.19±0.07	0.61±0.02	0.20±0.05	0.975	305	1.95
3,4- diMeOC_6_H_3_SO_2_Cl	25.0	40*^g^*	1.24±0.07	0.64±0.03	0.14±0.06	0.967	264	1.94
MeSO_2_Cl	45.0	39*^g^*	1.17±0.04	0.49±0.02	0.23±0.05	0.981	454	2.39
*i*-PrSO_2_Cl	45.0	19*^g^*	1.28±0.05	0.64±0.03	0.18±0.06	0.988	333	2.00
C_6_H_5_CH_2_SO_2_Cl	45.0	25*^g^*	0.80±0.06	0.39±0.04	0.21±0.06	0.947	95	2.05
(CH_3_) _2_NSO_2_Cl	25.0	32*^g^*	1.20±0.04	0.72±0.03	0.11±0.04	0.985	478	1.67
2-thiopheneSO_2_Cl	25.0	34*^g^*	1.35±0.05	0.70±0.02	0.28±0.05	0.983	455	1.93

a Number of data points.

b With associated standard error.

c Multiple correlation coefficient.

d *F*-test value.

e Omitting 90% and 87% acetone.

f Omitting 90-75% acetone.

g From tabulation in ref. [45]; where appropriate, references to earlier sources are given in this reference.

**Table 7.** The kinetic solvent isotope effect (KSIE) for solvolyses in methanol and methanol-*d* (*k*_MeOH_/*k*_MeOD_) and the ratios of specific rates for solvolyses in 40% (v/v) ethanol-water relative to 97% (w/w) TFE-water.

Substrate	*k*_MeOH_/*k*_MeOD_ (T ºC)	*k*_40EtOH_/*k*_97TFE_*^a^* (T ºC)
**1**	1.79 (25º)*^b^*	2900 (25º)*^c^*
**2**	2.31 (25º)*^b^*	15000 (25º)*^c^*
**3**	1.76 (45°)	846 (45°)
4-MeOC_6_H_4_SO_2_Cl	1.58 (25º)*^b^*	300 (25º)*^c^*
4-MeC_6_H_4_SO_2_Cl	1.72 (25º)*^b^*	450 (25º)*^c^*
MeSO_2_Cl	1.62 (25º); 1.51 (35º)*^d^*	2010 (45º)*^e^*
*i*-PrSO_2_Cl	2.54 (25º); 2.41 (35º)*^f^*	2790 (45º)*^g^*
(CH_3_)_2_NSO_2_Cl		359 (25º)*^h^*
4-MeO-2,6- diMeC_6_H_2_SO_2_Cl	1.58 (25º)*^b,^^i^*	89 (25º)*^i^*
2,4,6-triMeC_6_H_2_SO_2_Cl	1.68 (25º)*^b,^^j^*	202 (25º)*^j^*
3,4-diMeOC_6_H_3_SO_2_Cl	1.45 (25º)*^k^*	386 (25º)*^k^*

*_a_* Solvents with similar *Y*_Cl_ but very different *N*_T_ values.

b Values from ref. [19].

c Values from ref. [22]; references for values determined elsewhere are given.

d Values from ref. [48].

e Value for 40% EtOH from ref. [48] and value for 97% TFE from ref. [50].

f Values from ref. [49].

g Values from ref. [21], value for 40% EtOH of 108 × 10^−6^ s^−1^ obtained by interpolation.

h Value for 40% EtOH of 733 × 10^−6^ s^−1^ obtained from Arrhenius plots of data in ref. [25] and value for 97% TFE from ref. [21].

i Values from ref. [29].

j Values from ref. [18].

k Values from ref. [31].
